# Higher risk of carotid plaque among lean individuals with non-alcoholic fatty liver disease: A retrospective study

**DOI:** 10.1371/journal.pone.0316997

**Published:** 2025-02-03

**Authors:** Jiangfeng Xue, Lun Zhao, Liang Shao, Huiwang Zhang, Yewei Feng, Ping Shuai

**Affiliations:** 1 Department of Health Management, The People’s Hospital of Yubei District of Chongqing, Chongqing, China; 2 Department of Digestive System Disease, The People’s Hospital of Yubei District of Chongqing, Chongqing, China; 3 Department of Sohome Health Management, Sichuan Academy of Medical Sciences & Sichuan Provincial People’s Hospital, Chengdu, Sichuan Province, China; 4 Department of Health Management, Sichuan Academy of Medical Sciences & Sichuan Provincial People’s Hospital, Chengdu, Sichuan Province, China; Kaohsiung Medical University Hospital, TAIWAN

## Abstract

**Background:**

Lean individual with non-alcoholic fatty liver disease (L-NAFLD) is a prominent area of research, yet its pathogenesis and association with other diseases such as atherosclerotic cardiovascular disease remain uncertain.

**Object:**

A retrospective study, investigate the association between non-alcoholic fatty liver disease (NAFLD) and carotid plaque (CP) in lean [body mass index (BMI) <24Kg/m^2^] and non-lean (BMI≥24Kg/m^2^) populations, as well as identify the related influence factors.

**Method:**

3,587 participants were eligible and categorized into 4 groups based on the presence with CP and BMI, binary logistic regression analysis was utilized alongside other statistical methods.

**Results:**

L-NAFLD participants had a 1.395-fold higher risk of CP compared to lean individuals without NAFLD. Age, gender, systolic blood pressure, low-density lipoprotein cholesterol, fasting blood glucose, and Fibrosis-4 index (FIB-4) were identified as independent risk factors with cutoff values lower than the normal upper limits. However, this association was not observed among non-lean participants, regardless of confounding factors adjustment. Moreover, the impact of FIB-4 on the association of NAFLD and CP was more significant in lean CP participants (OR = 1.360 for 1.30 ~ 2.67, and OR = 2.002 for >2.67~<3.48) than in non-lean CP ones.

**Conclusion:**

The L-NAFLD population had a higher risk of CP, while lean CP individuals experienced more severe liver fibrosis. Implementing stricter management of risk factors may improve the health status of high-risk populations.

## Introduction

The high prevalence of non-alcoholic fatty liver disease (NAFLD) makes it the predominant liver disease globally and in China [1], and lean individuals, whose body mass index (BMI) falls within the lower or normal range of values, also exhibit a significant prevalence of NAFLD, approximately 5.1% in the general population and 19.2% among NAFLD patients [2]. NAFLD not only poses risks to people’s health conditions but also imposes significant economic burdens [3,4]. Accumulating evidence suggests that it is closely associated with various systemic diseases, including type 2 diabetes [5], atherosclerotic cardiovascular disease (ASCVD) [6], chronic kidney disease [7], and digestive system tumors [8,9]. Consequently, our focus shift from liver injury to multi-organ damage.

In terms of the association between NAFLD and ASCVD, numerous studies suggest that both conditions may share similar pathogenic mechanisms, such as insulin resistance (IR), low-grade inflammation, dysbiosis of gut microbiota [10]. While previous studies primarily focused on general populations, the advancement of recognition has led to an increasing subgroup studies [11,12]. Despite extensive research and hypotheses, the underlying pathogenesis of lean non-alcoholic fatty liver disease (L-NAFLD), as well as its mechanisms of association with ASCVD remain elusive [13,14], with certain research findings even exhibit contradictory outcomes [5].

To address these inquiries, our aim is to investigate the association between NAFLD and carotid plaque (CP, a subclinical manifestation of ASCVD [15]) in both lean and non-lean populations as part of subgroup analysis, along with the influence factors, thereby providing novel evidence for comprehending the association between these two conditions.

## Materials and method

### Study population

A retrospective study, enrolled a total of 33,160 individuals (from 01/01/2023 to 31/12/2023) who underwent physical examinations in the Department of health management, The People’s Hospital of Yubei District, Chongqing. This study had been approved by the ethics committee of The People’s Hospital of Yubei District of Chongqing (approval number: 2024C02) and the original data we accessed on 14/09/2024.

**Fig 1:**
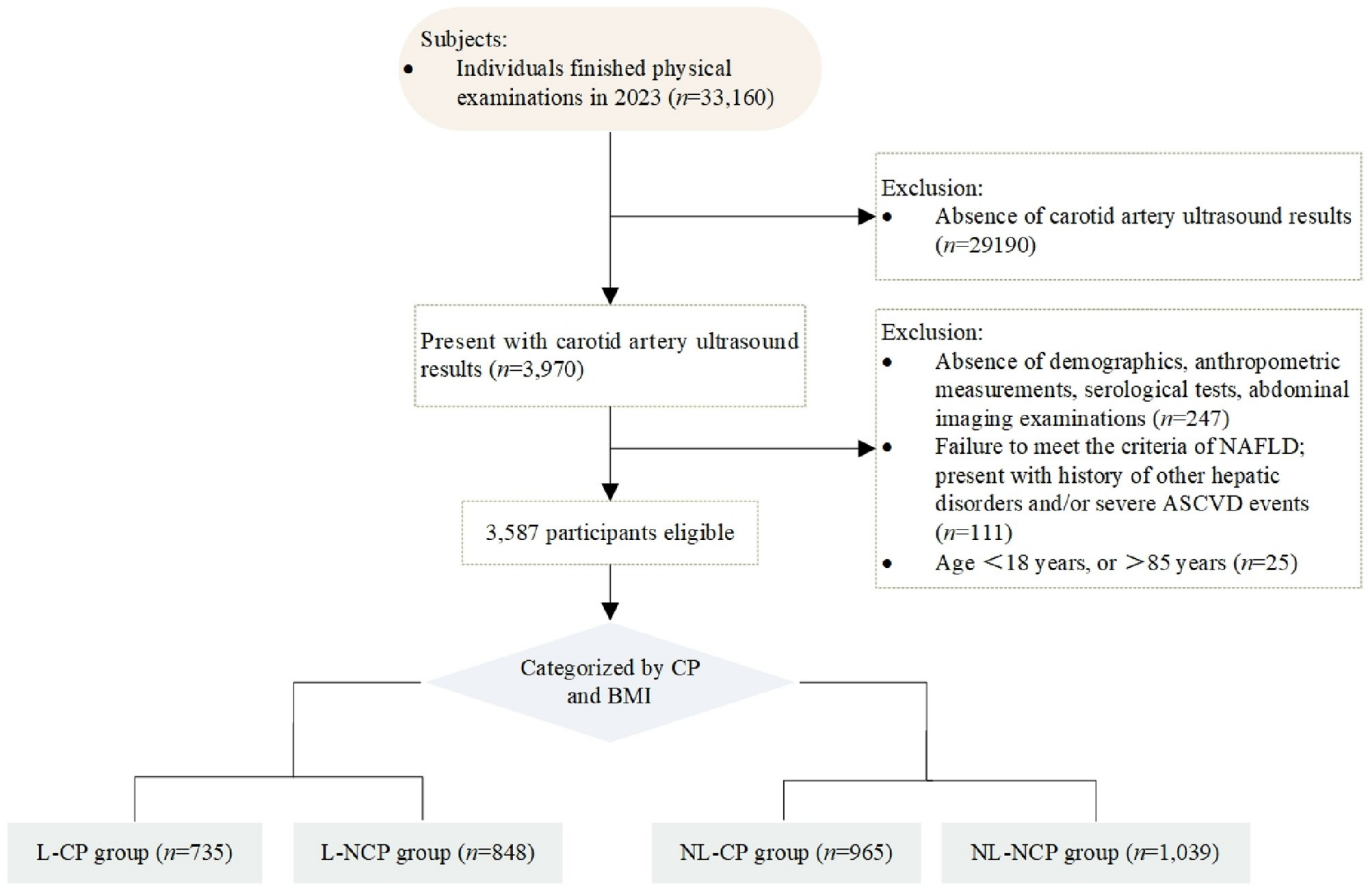
Flowchart of study population and group definitions. Abdominal imaging examinations: Ultrasound, computerized tomography, magnetic resonance imaging. Other hepatic disorders: Hepatic cirrhosis, hepatocellular carcinoma, etc. Severe ASCVD events: Myocardial infarction, post-coronary artery bypass grafting, and post-stent implantation, etc. NAFLD: Non-alcoholic fatty liver disease; ASCVD: Atherosclerotic cardiovascular disease; CP: Carotid plaque; BMI: Body mass index; L-CP: BMI <24 Kg/m^2^ and present with CP; L-NCP: BMI <24 Kg/m^2^ but absent with CP; NL-CP: BMI≥24 Kg/m^2^ and present with CP; NL-NCP: BMI≥24 Kg/m^2^ and absent with CP.

The participants inclusion criteria: 1) aged between 18 and 85 years; 2) complete data on demographics, anthropometric measurements, serological tests, carotid artery ultrasound results and abdominal imaging examinations. And the exclusion criteria: 1) the participants did not meet the diagnostic criteria for NAFLD according to Chinese guidelines, despite being identified with fatty liver disease (FLD) through abdominal imaging examinations [16]. 2) with history of other hepatic disorders such as hepatic cirrhosis, hepatocellular carcinoma; and 3) with a history of severe ASCVD events, such as myocardial infarction, post-coronary artery bypass grafting, and post-stent implantation. Finally, 3587 participants were eligible (Fig 1). What needs to be clarified is that for the participant who underwent multiple physical examinations, the initial results meeting the study requirements were extracted solely by the workstation system engineer.

### Group definitions

CP is an inflammatory vascular disease driven by lipids, characterized by a focal structural protrusion into the artery lumen > 0.5 mm or 50% of the surrounding intima-media thickness (IMT) value, or exhibiting an IMT >1.5 mm measured from the media-adventitia interface to the intima-lumen interface [17]. The classification of CP and non-CP (NCP) was based on the results of carotid artery ultrasound. BMI is computed by dividing weight by the square of height (Kg/m^2^). We employed Chinese BMI criteria [18], the value <24Kg/m^2^ was classified as lean (L), while ≥24Kg/m^2^ was categorized as non-lean (NL). Based on carotid artery ultrasound results and BMI values, all participants were divided into 4 groups: L-CP, L-NCP, NL-CP and NL-NCP (Fig 1).

### Clinical characteristics and outcomes

The demographics were collected through ID card scanning, medical history information was obtained by doctors. Physical examinations were conducted under fasting conditions more than 6 hours. Participants were required with light clothes and be barefoot for the anthropometric measure, including height, weight, and blood pressure (BP), which performed in a quiet examination room using automated instruments (SH-V5, SHANGHE, Zhengzhou, China, for height and weight measurement; HBP-9030, OMRON Corp., Kyoto, Japan, for BP measurement) and under the supervision of trained nurses. The measurements were accurate to 0.1cm for height, 0.1Kg for weight, and 1mmHg for BP.

Serological tests included: platelet count (PLT), hemoglobin level (HB), alanine aminotransferase (ALT), aspartate aminotransferase (AST), gamma-glutamyl transferase (GGT), triglyceride (TG), total cholesterol (TC), high density lipoprotein cholesterol (HDL-c), low density lipoprotein cholesterol (LDL-c), fasting blood glucose (FBG), creatinine (CRE), blood urea nitrogen (BUN) and uric acid (UA). The blood samples were collected by experienced nurses and tested in our hospital’s medical laboratory adhering to the standard operating procedures.

Fibrosis-4 index (FIB-4) is a noninvasive data model utilized for assessing the extent of liver fibrosis, the equation is: [age (year)×AST (U/L)] / [PLT (×10^9^/L)×ALT (U/L)^1/2^], scores of <1.30, 1.30~2.67, >2.67~<3.48 and ≥3.48 respectively indicate the absence, presence, advanced liver fibrosis and hepatic cirrhosis [19].

The diagnosis criteria for NAFLD according to the 2018 Chinese guideline includes: 1) FLD diagnosed through abdominal imaging examinations or liver biopsy; 2) eliminating other hepatic disorder (such as alcoholic liver disease, genotype 3 HCV infection, autoimmune hepatitis, hepatic steatosis) and disease may cause FLD (medications utilization, total parenteral nutrition, inflammatory bowel disease, celiac disease, hypothyroidism syndrome of Cushing’s syndrome, β-lipoprotein deficiency syndrome, lipodystrophic diabetes mellitus, Mauriac syndrome, etc.); 3) absence of alcohol consumption, means ≤210 g/week for male, ≤140 g/week for female [16].

The aspect requires clarification was that the abdominal imaging examinations in our study included ultrasound, computerized tomography (CT, Brilliance iCT, PHILIPS, Philips Electronics Ltd., Netherlands) and magnetic resonance imaging (MRI, Vantage Elan^™^ 1.5T, Canon, Canon Inc., Tokyo, Japan). Both carotid artery and abdominal ultrasound examinations were performed in our department using the EPIQ 7 system (PHILIPS, Philips Ultrasound Inc, Netherlands), while CT and MRI scans were conducted in the radiology department of our hospital. Furthermore, all information and results underwent double verification for accuracy.

### Statistical analysis

Categorical variables were expressed as *n* (*%*) and compared by the Chi-square test. Continuous variables were analyzed by Kolmogorov-Smirnov test for distribution. Normally distributed continuous variables were described by mean±$\bar{S}$ and T-test was utilized for comparison between groups. While abnormal distributed continuous variables were described by median [interquartile range, (IQR)], and compared by Mann-Whitney U test between groups.

Binary logistic regression analysis was utilized to investigate the association between NAFLD and CP in both lean and non-lean participants, as well as the influence of FIB-4 on the association. Continuous variables were transformed into categorical variables to narrow down the range of risk factors. We developed multiple predictive models to assess the risks of CP, and then, plotted receiver operating characteristic (ROC) curves and calculated the areas under the curve (AUC) to evaluate the reliability of models. Subsequently, the optimal cutoff values of the risk factors were calculated. Data processing, analysis and figure creation were performed ultilizing SPSS Statistics Version 22 (IBM Corp., Armonk, NY, USA), R 4.3.0 version (R Foundation for Statistical Computing; Vienna, Austria), and Zstats v1.0 (www.zstats.net). *P*< 0.05 was considered statistically significant.

## Results

### Baseline characteristics

A total of 3,587 participants [2,023 (56.4%) were male] were eligible out of the initial pool of 33,160 individuals, with median age 56 years old, 1,583 (44.1%) were lean individuals. Participants with CP, NAFLD accounted for 1,700 (47.4%), 1,877 (52.3%), respectively (Tables 1 and S1).

**Table 1:** Baseline characteristics of 4 groups and comparison in lean and non-lean population.

Characteristics	Total (3587)	Lean			Non-lean		
		CP [735(20.5)]	NCP [848(23.6)]	*P*	CP [965(26.9)]	NCP [1039(29.0)]	*P*
Demographics							
Age (year)	56 (15)	61 (15)	52 (13)	**<0.001** ^a^	60 (17)	52 (12)	**<0.001**
18~39 [*n* (*%*)]	247 (6.9)	10 (1.4)	112 (13.2)	**<0.001**	13 (1.3)	112 (10.8)	**<0.001**
40~64 [*n* (*%*)]	2468 (68.8)	415 (56.4)	646 (76.2)	NA	577 (59.8)	830 (79.9)	NA
65 [*n* (*%*)]	872 (24.3)	310 (42.2)	90 (10.6)	NA	375 (38.9)	97 (9.3)	NA
Gender [*n* (*%*)]				**<0.001**			0.104
Male	2023 (56.4)	393 (53.5)	335 (39.5)	NA	641 (66.4)	654 (62.9)	NA
Female	1564 (43.6)	342 (46.5)	513 (60.5)	NA	324 (33.6)	385 (37.1)	NA
Anthropometric measure							
Height (cm)	162.0 (12.4)	160.5 (13.0)	160.0 (11.0)	0.512	162.5 (11.8)	163.5 (12.5)	**0.024**
Weight (Kg)	64.0 (15.6)	56.0 (11.3)	56.00 (9.8)	0.355	70.0 (12.4)	71.0 (13.3)	**0.018**
SBP (mmHg)	130 (23)	132 (24)	121 (24)	**<0.001**	136 (24)	129 (20)	**<0.001**
DBP (mmHg)	78 (16)	76 (15)	74 (15)	**<0.001**	80 (15)	80 (16)	0.291
BMI (Kg/m^2^)	24.4 (4.1)	22.23 (2.30)	22.26 (2.20)	0.498	26.26 (2.71)	26.36 (2.82)	0.318
Serological tests							
PLT (×10^9^/L)	199.2 (66.9)	195.3 (67.7)	202.0 (63.7)	0.075	195.6 (66.6)	203.5 (70.4)	**<0.001**
HB (g/L)	141.2 (19.6)	138.1±13.6	136.6 (18.1)	0.232	143.4 (18.7)	146.0 (19.7)	**<0.001**
ALT (U/L)	20.0 (12.6)	16.9 (9.5)	17.1 (10.3)	0.787	21.4 (12.8)	23.7 (16.6)	**<0.001**
AST (U/L)	22.7 (7.2)	22.7 (6.7)	21.9 (6.8)	**0.007**	22.7 (7.0)	23.6 (8.2)	**0.010**
GGT (U/L)	24.4 (22.3)	20.9 (16.1)	19.2 (14.2)	**<0.001**	28.6 (23.6)	29.9 (26.8)	0.178
TG (mmol/L)	1.41 (1.03)	1.19 (0.76)	1.15 (0.74)	0.086	1.6 (1.1)	1.7 (1.2)	0.121
TC (mmol/L)	4.9 (1.3)	5.0±1.0	4.8 (1.1)	**0.004**	5.0±1.0	4.9 (1.3)	0.505
HDL-c (mmol/L)	1.6 (0.5)	1.7 (0.5)	1.7 (0.6)	0.479	1.5 (0.5)	1.5 (0.5)	0.798
LDL-c (mmol/L)	2.59 (0.88)	2.61±0.70	2.45 (0.79)	**<0.001**	2.69±0.70	2.63 (0.79)	0.484
FBG (mmol/L)	5.31 (0.89)	5.28 (0.92)	5.08 (0.68)	**<0.001**	5.54 (1.24)	5.36 (0.83)	**<0.001**
CRE (μmol/L)	67.9 (21.3)	66.3 (20.4)	63.0 (19.7)	**<0.001**	70.9 (20.1)	70.4 (21.6)	0.118
BUN (mmol/L)	5.3 (1.8)	5.5 (1.9)	5.2 (1.7)	**<0.001**	5.4 (1.8)	5.3 (1.7)	**<0.001**
UA (μmol/L)	336.2 (121.7)	312.9 (104.9)	304.1 (112.0)	0.125	356.8 (121.4)	362.7 (124.3)	0.378
FIB-4	1.41 (0.90)	1.72 (0.99)	1.34 (0.81)	**<0.001**	1.55 (0.98)	1.24 (0.71)	**<0.001**
<1.30 [*n* (*%*)]	1485 (41.4)	183 (24.9)	399 (47.1)	**<0.001**	335 (34.7)	568 (54.7)	**<0.001**
1.30~2.67 [*n* (*%*)]	1796 (50.1)	439 (59.7)	404 (47.6)	NA	511 (53.0)	442 (42.5)	NA
>2.67~<3.48 [*n* (*%*)]	209 (5.8)	72 (9.8)	32 (3.8)	NA	82 (8.5)	23 (2.2)	NA
≥3.48 [*n* (*%*)]	97 (2.7)	41 (5.6)	13 (1.5)	NA	37 (3.8)	6 (0.6)	NA
NAFLD [*n* (*%*)]				**<0.001**			0.757
Presence	1877 (52.3)	223 (30.3)	188 (22.2)	NA	709 (73.5)	757 (72.9)	NA
Absence	1710 (47.7)	512 (69.7)	660 (77.8)	NA	256 (26.5)	282 (27.1)	NA

^a^The bold numbers signify statistical significance.

BMI: body mass index; Lean: BMI <24 Kg/m^2^; non-Lean: BMI≥24 Kg/m^2^; CP: Carotid plaque; NCP: Absent with CP; SBP: Systolic blood pressure; DBP: Diastolic blood pressure; PLT: Platelet count; HB: Hemoglobin; ALT: Alanine aminotransferase; AST: Aspartate aminotransferase; GGT: Gamma-glutamyl transferase; TG: Triglyceride; TC: Total cholesterol; HDL-c: High density lipoprotein cholesterol; LDL-c: Low density lipoprotein cholesterol; FBG: Fasting blood glucose; CRE: Creatinine; BUN: Blood urea nitrogen; UA: Uric acid; FIB-4: Fibrosis-4 index; NAFLD: Non-alcoholic fatty liver disease.

In the lean population, L-CP group[735 (20.5%)] was characterized by older age (61 years vs 52 years, *P*<0.001) and higher proportion of males (53.5% vs 39.5%, *P*<0.001) compared to L-NCP group. Additionally, there were significant differences in BP, TC (5.0 mmol/L vs 4.8 mmol/L, *P* = 0.004), LDL-c (2.61mmol/L vs 2.45mmol/L, *P*<0.001), FBG (5.28 mmol/L vs 5.08 mmol/L, *P*<0.001), FIB-4 (1.72 vs 1.34, *P*<0.001) and the distribution of NAFLD (30.3% vs 22.2%, *P*<0.001).

In the non-lean population, NL-CP group exhibited a higher median age compared to NL-NCP group (60 years vs 52 years, *P*<0.001), along with significant difference of SBP (136 mmHg vs 129 mmHg, *P*<0.001), FBG (5.54 mmol/L vs 5.36 mmol/L, *P*<0.001), and FIB-4 (1.55 vs 1.24, *P*<0.001). However, there were no significant differences observed in terms of gender, lipids and the distribution of NAFLD (Table 1).

### Distribution of CP in lean and non-lean NALFD participants

**Fig 2:**
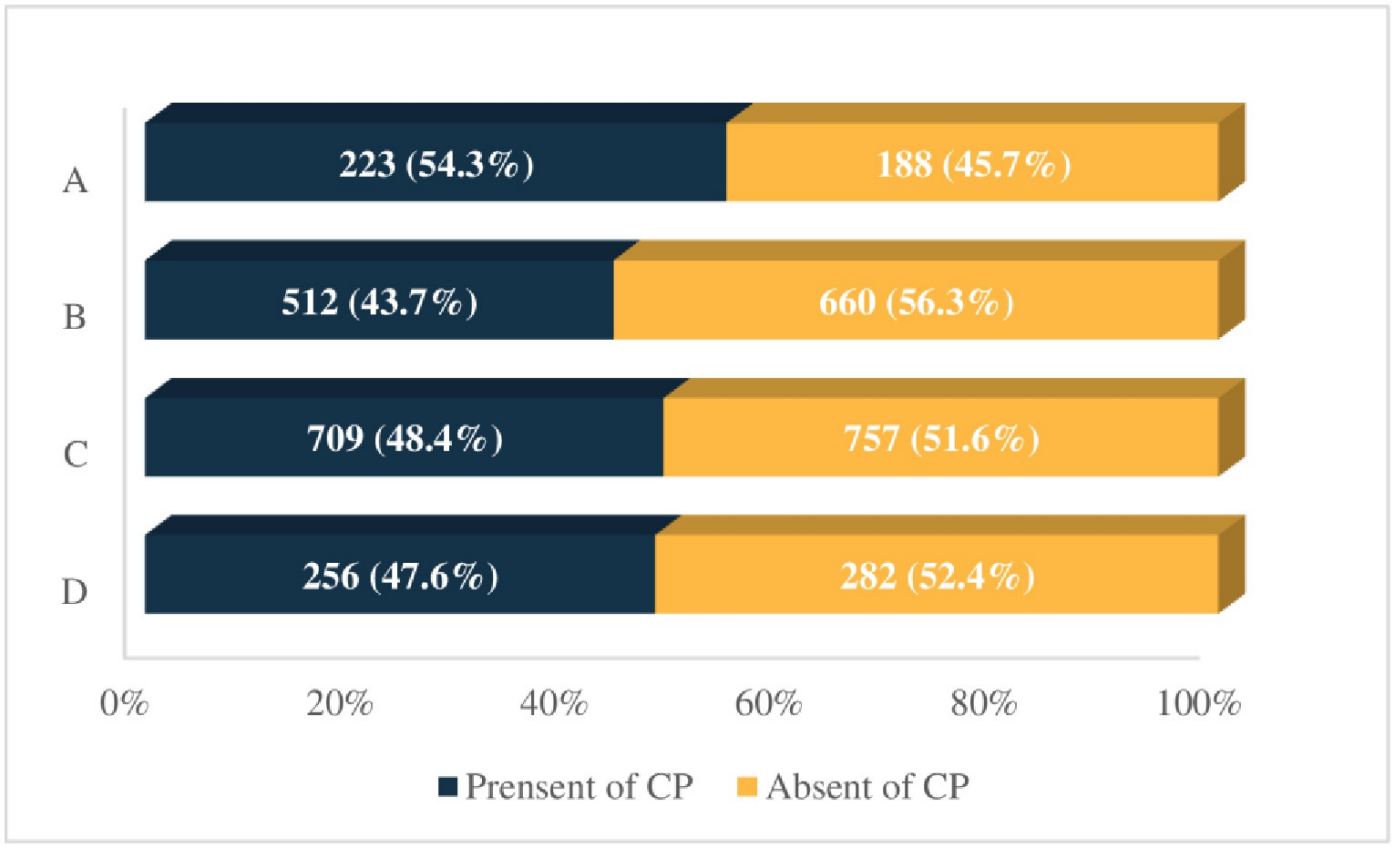
Distribution of CP across various BMI and NAFLD combinations. A & B were lean participants present & absent with NAFLD, and *χ*^*2*^ = 13.673, *P*<0.001. C & D were non-lean participants present & absent with NAFLD, and *χ*^*2*^ = 0.096, *P* = 0.757. BMI: Body mass index; NAFLD: Non-alcoholic fatty liver disease; CP: Carotid plaque.

The L-NAFLD participants exhibited the highest proportion of CP (54.3%), which was similar to the baseline characteristics, followed by non-lean participants, while the lowest proportion was observed in the lean but absent with NAFLD (L-NNAFLD) group (43.7%). In contrast to lean individuals (*χ*^*2*^ = 13.673, *P*<0.001), there was no significant difference of CP distribution among non-lean individuals (*χ*^*2*^ = 0.096, *P* = 0.757) (Fig 2).

### Prediction models of CP in lean and non-lean NAFLD participants

The lean and non-lean groups were analyzed separately using binary logistic regression analysis to develop risk prediction models for CP. The Crude model was adjusted for NAFLD solely; Model 1 was additionally adjusted for age and gender based on the crude model; Model 2 incorporated further adjustments for SBP, BMI, TG, LDL-c, FBG, and UA based on Model 1; and finally, Model 3 integrated FIB-4 adjustment based on Model 2. The results demonstrated a significant association between NAFLD and CP in the lean participants even after adjusted confounding variables (*P*<0.05). However, in the non-lean participants, no association was observed, regardless of variables were adjusted or not (Table 2).

**Table 2:** Prediction models of CP in lean and non-lean NAFLD participants.

Model^a^	L-CP and L-NCP groups						NL-CP and NL-NCP groups					
	B	Wald	OR	95% CI		*P*	B	Wald	OR	95% CI		*P*
				Lower	Upper					Lower	Upper	
Crude	0.425	13.587	1.529	1.220	1.916	**<0.001** ^b^	0.031	0.096	1.032	0.847	1.257	0.757
Model 1	0.280	4.918	1.323	1.033	1.693	**0.027**	0.182	2.634	1.199	0.963	1.493	0.105
Model 2	0.293	4.220	1.340	1.014	1.772	**0.040**	0.147	1.520	1.159	0.917	1.465	0.218
Model 3	0.333	5.381	1.395	1.053	1.848	**0.020**	0.154	1.641	1.166	0.922	1.476	0.200

^a^Crude model adjusted NAFLD; Model 1 adjusted age and gender based on Crude model, Model 2 adjusted body mass index (BMI), systolic blood pressure, triglyceride, low density lipoprotein cholesterol, fasting blood glucose and uric acid based on Model 1, Model 3 adjusted Fibrosis-4 index based on Model 2.

^b^The bold numbers signify statistical significance.

L-CP: BMI <24 Kg/m^2^ and present with carotid plaque (CP); L-NCP: BMI <24 Kg/m^2^ but absent with CP; NL-CP: BMI≥24 Kg/m^2^ and present with CP; NL-NCP: BMI≥24 Kg/m^2^ but absent with CP; OR: Odds ratio; CI: Confidence interval.

### ROC curves for models predicting the risk of CP in L-NAFLD participants

**Fig 3:**
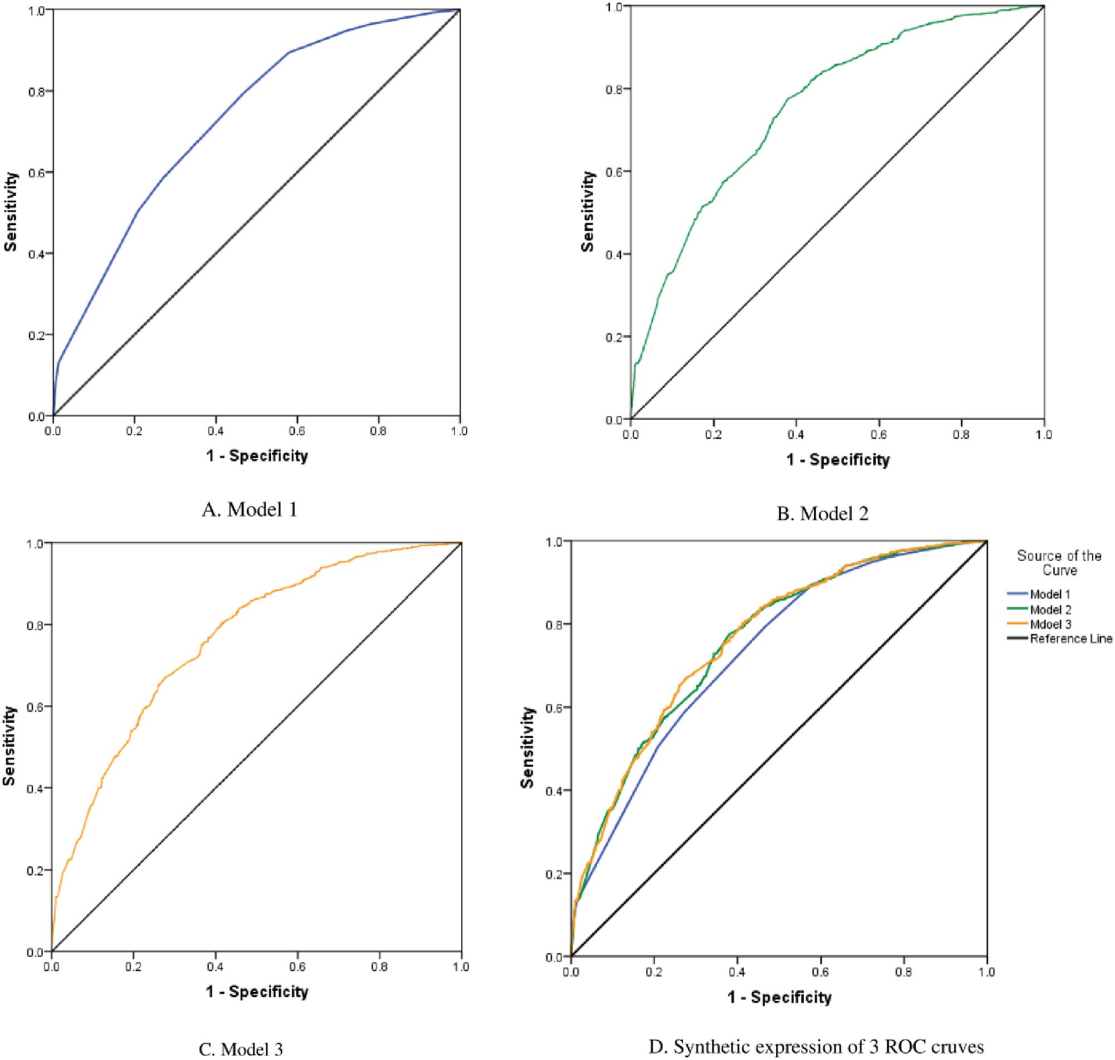
ROC curves for models predicting the risk of CP in L-NAFLD participants. (A) ROC curve for Model 1; (B) ROC curve for Model 2; (C) ROC curve for Model 3. (D) synthetic expression of 3 models. AUC of 3 curves was 0.731, 0.758, 0.763, respectively. ROC curve: Receiver operating characteristic curves; AUC: Area under the curve.

Subsequently, ROC curves were plotted for 3 models predicting the risk of CP in L-NAFLD participants (Fig 3). The corresponding AUC values were 0.731, 0.758, and 0.763, respectively.

### The influence factors on the association between NAFLD and CP

**Fig 4:**
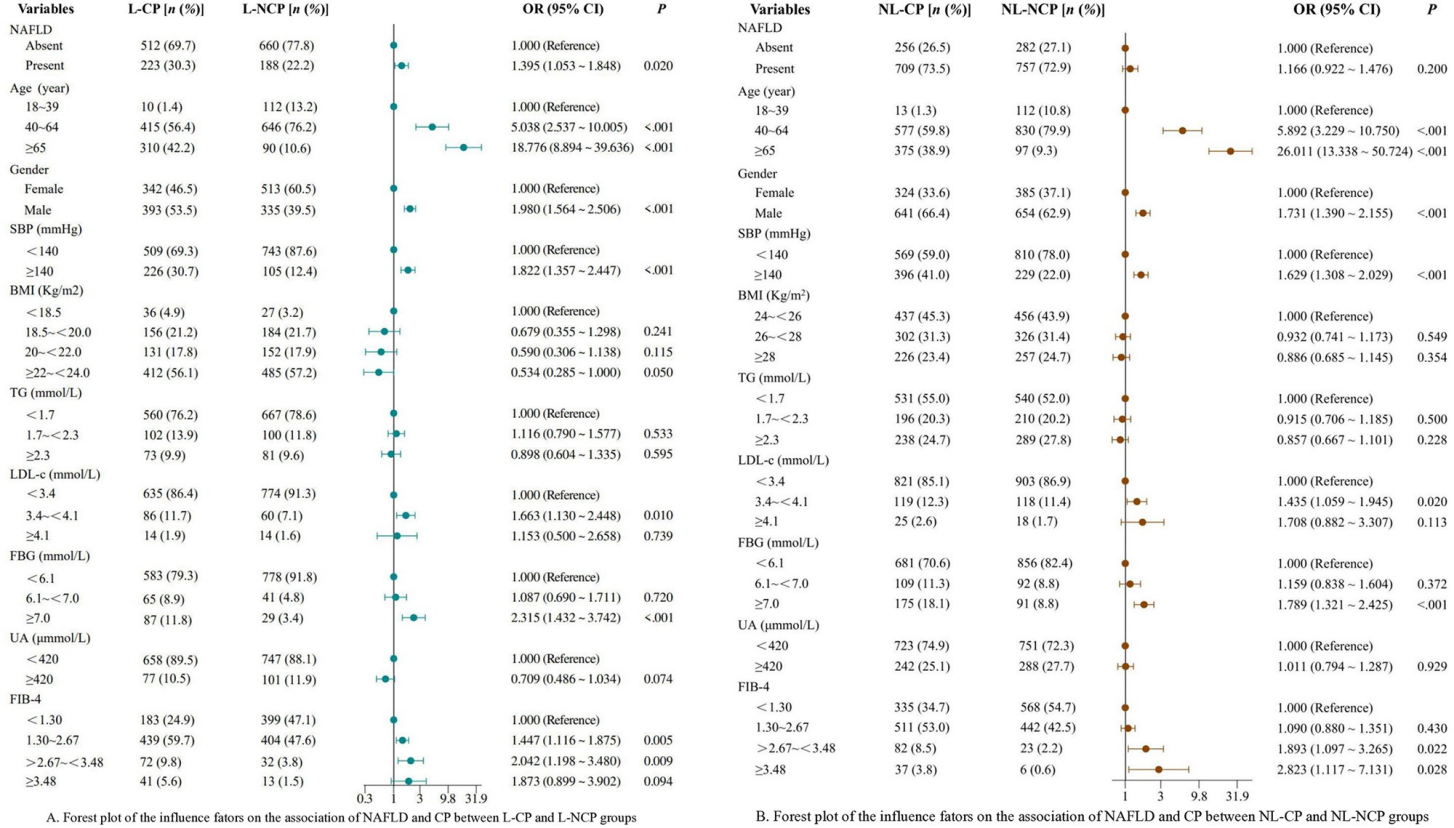
Forest plots of the influence factors on the association of NAFLD and CP in lean and non-lean participants. (A) Analysis between L-CP and L-NCP groups, NAFLD was related to CP, along with age, gender, SBP, LDL-c, FBG and FIB-4 were identified as independent risk factors. (B) Analysis between NL-CP and NL-NCP groups, the association of NAFLD and CP was not observed, though other risk factors were similar with lean participants. OR: Odds ratio; CI: Confidence interval; NAFLD: Non-alcoholic fatty liver disease; CP: Carotid plaque; SBP: Systolic blood pressure; BMI: Body mass index; TG: Triglyceride; LDL-c: Low density lipoprotein cholesterol; FBG: Fasting blood glucose; UA: Uric acid; FIB-4: Fibrosis-4 index; L-CP: BMI <24 Kg/m^2^ and present with CP; L-NCP: BMI <24 Kg/m^2^ but absent with CP; NL-CP: BMI≥24 Kg/m^2^ and present with CP; NL-NCP: BMI≥24 Kg/m^2^ but absent with CP.

The association between NAFLD and CP was observed in lean participants. L-NAFLD participants had a 1.395-fold higher risk of CP compared to L-NNAFLD (95% CI: 1.053~1.848, *P* = 0.020), participants aged 40 years or above had significantly increased risk (OR = 5.038, 95% CI: 2.537~10.005 for age 40 ~ 64 years, *P*<0.001; OR = 18.776, 95% CI: 8.894 ~ 39.636 for age≥65 years, *P*<0.001), as well as males (OR = 1.980, 95% CI: 1.564 ~ 2.506, *P*<0.001), SBP ≥140mmHg (OR = 1.822, 95% CI: 1.357 ~ 2.477, *P*<0.001), LDL-c: 3.4mmol/L ~ <4.1mmol/L (OR = 1.663, 95% CI: 1.130 ~ 2.448, *P* = 0.010), FBG≥7.0mmol/L (OR = 2.315, 95% CI: 1.432 ~ 3.742, *P*<0.001), and FIB-4 ≥1.30 (OR = 1.447, 95% CI: 1.116 ~ 1.875 for 1.3 ~ 2.67, *P* = 0.005; OR = 2.042, 95% CI: 1.198 ~ 3.480 for >2.67 ~ <3.48, *P* = 0.009) were identified as independent risk factors (Fig 4A). However, the association between NAFLD and CP was not observed in non-lean participants, even after adjusting confounding factors, while other risk factors were similar with lean participants (Fig 4B).

### The cutoff values of risk factors associated with L-NAFLD and CP

The cutoff values of risk factors were presented in Table 3, and the corresponding thresholds were determined as follows: 54.5 years for age, 131 mmHg for SBP, 2.82 mmol/L for LDL-c, 5.34 mmol/L for FBG, and 1.42 for FIB-4.

**Table 3:** The cutoff values of risk factors associated with L-NAFLD and CP.

Variables	AUC	95% CI		*P*	Cutoff value	Normal limits	Youden’s index
		Lower	Upper				
Age (year)	0.761	0.738	0.784	**<0.001** ^a^	54.5	NA	0.386
SBP (mmHg)	0.669	0.642	0.696	**<0.001**	131	90~140	0.256
LDL-c (mmol/L)	0.554	0.525	0.582	**<0.001**	2.82	<3.4	0.127
FBG (mmol/L)	0.617	0.590	0.645	**<0.001**	5.34	3.8~6.1	0.180
FIB-4	0.657	0.631	0.684	**<0.001**	1.42	<1.30	0.238

^a^The bold numbers signify statistical significance.

AUC: Area under the curve; CI: Confidence interval; SBP: Systolic blood pressure; LDL-c: Low density lipoprotein cholesterol; FBG: Fasting blood glucose; FIB-4: Fibrosis-4 index. L-NAFLD: Body mass index < 24 Kg/m^2^ and with non-alcoholic fatty liver disease; CP: Carotid plaque.

### The influence of FIB-4 on the association of NAFLD and CP

**Fig 5:**
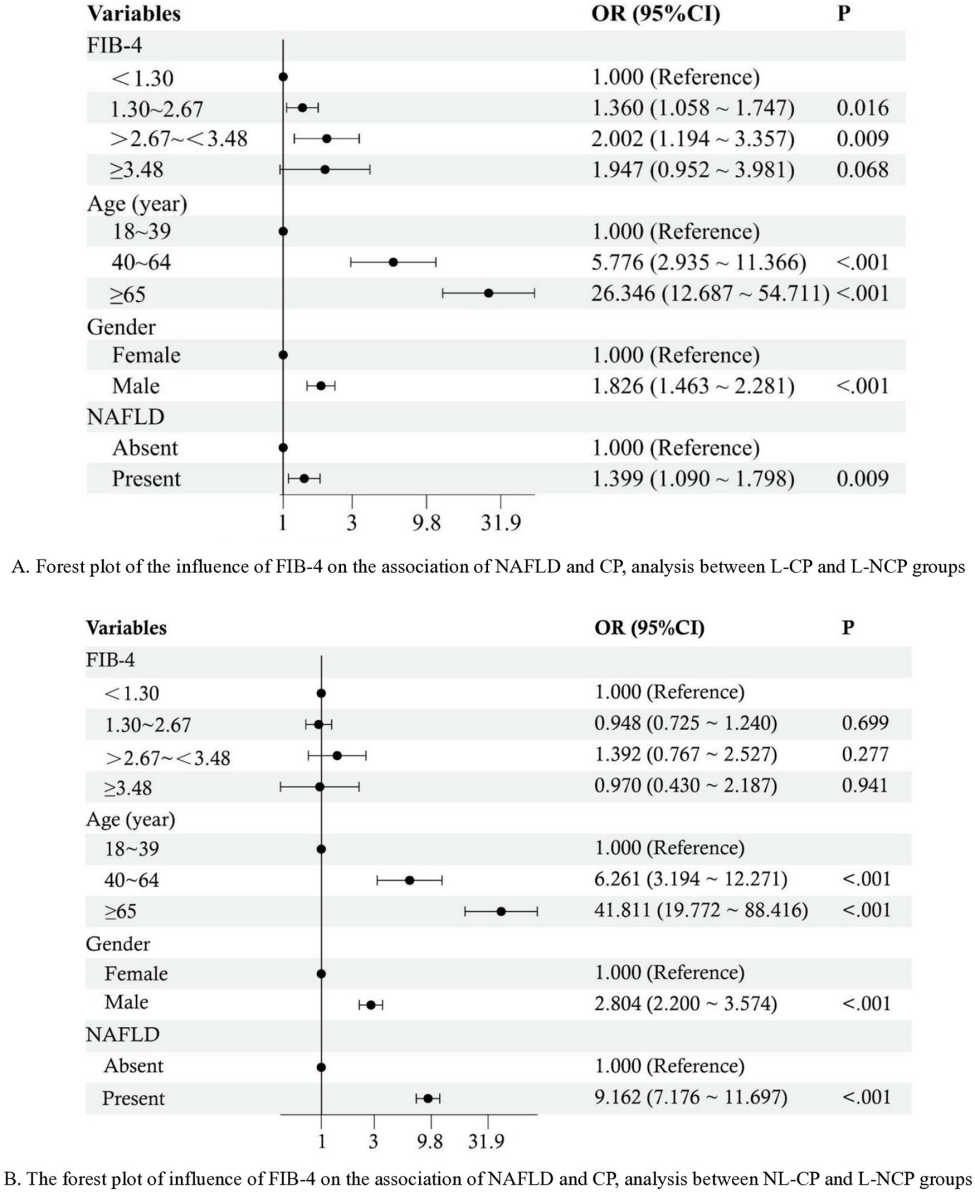
The forest plots of the influence of FIB-4 on the association of NAFLD and CP. (A) Analysis between L-CP and L-NCP groups. (B) Analysis between NL-CP and L-NCP groups. OR: Odds ratio; CI: Confidence interval; NAFLD: Non-alcoholic fatty liver disease; CP: Carotid plaque; FIB-4: Fibrosis-4 index; L-CP: Body mass index (BMI) <24 Kg/m^2^ and present with CP; L-NCP: BMI <24 Kg/m^2^ but absent with CP; NL-CP: BMI≥24 Kg/m^2^ and present with CP.

To investigate the differential impact of FIB-4 on the association between NAFLD and CP in different BMI populations, we designated the L-NCP group as the reference category. The plots demonstrated that FIB-4 was an independent risk factor of CP in L-NAFLD participants after adjusting age and gender (OR = 1.360, 95% CI: 1.058 ~ 1.747 for 1.30 ~ 2.67, *P* = 0.016; OR = 2.002, 95% CI: 1.194 ~ 3.357 for >2.67~<3.48, *P* = 0.009; Fig 5A). Conversely, no influence was observed from the analysis between NL-CP and L-NCP groups, even after adjusting for age and gender (Fig 5B).

## Discussion

This study focuses on investigating the association between NAFLD and CP in lean and non-lean populations, which is limited explored [20]. Our finding revealed the association exhibited in the lean participants but not in the non-lean individuals. To further validate the result, we compared the proportion of CP across various BMI and NAFLD combinations, which demonstrated that L-NAFLD participants exhibited the highest rate of CP, similar with the previous results. The potential factors contributing to the disparity in association maybe as follows.

The first noteworthy finding, which we also consider to be one of the highlights in our research, is the level and the impact of FIB-4, a valuable and widely utilized tool for assessing liver fibrosis and exhibits a positive correlation with the liver fibrosis [1,19]. In terms of baseline characteristics, we observed that the L-CP group exhibited the highest levels of FIB-4, and initial logistic regression analysis indicated that FIB-4 was an independent risk factor for CP. Therefore, we hypothesized that the L-CP participants had more severe liver fibrosis compared to the NL-CP ones. To validate this hypothesis and comprehend whether FIB-4’s impact on the association between NAFLD and CP was consistent across different BMI populations, we further analyzed both the L-CP and NL-CP groups with reference to the L-NCP group. The final analysis results consistently demonstrated a more significant influence of FIB-4 on the L-CP group. Meanwhile, the positive association between liver fibrosis and ASCVD has been supported [21], indicating that an elevated FIB-4 level likely indicates an increased risk of ASCVD [22]. Based on our findings and previous studies [23,24], we consider the heightened severity of liver fibrosis in L-NALFD participants contributes to the increased susceptibility to CP, with FIB-4 serving as an potential indicator. Due to the infrequency of liver biopsy in physical examinations, and the potential influence of geographical, ethnic, and methodological factors on research outcomes [23,25], we anticipate further investigations.

The second one is gender distribution. Not like in non-lean participants, L-CP group had a higher proportion of males compared to the L-NCP group. A recent multicenter cross-sectional study conducted in China revealed that men exhibit elevated rates of IMT, plaque, and atherosclerosis compared to women [26]. In the context of NAFLD, male is also a risk factors [27]. Moreover, in China, females shoulder a greater burden of household chores, exhibit heightened awareness towards energy intake, and engage less frequently in social dining, these maybe also reasons attributed to the difference.

The third one, though the mechanisms underlying the development of L-NAFLD and its interaction with ASCVD remain elusive [13,23,28], alongside limit subgroup researches [20], our analysis of existing data suggests that genetic phenotypes and sarcopenia may provide explanations for the association observed in different BMI populations:

1) The genotype: rs58542926 in TM6SF2 (transmembrane 6 superfamily member 2) and rs738409 in PNPLA3 (patatin-like phospholipase domain-containing protein 3) [23,28]. The polymorphism of the TM6SF2 gene plays an important role in liver and cholesterol metabolism [29], with the C>T variant being prevalent in lean FLD population [30,31]. The rs738409 has been identified as an independent factor associated with a higher risk of NAFLD and fibrosis in lean individuals [5], and GG variant of rs738409 can encode adiponutrin, which affects lipid metabolism. Considering the association of these gene products with hepatic injury and lipid metabolism, as well as the abnormal lipid metabolism being a contributing factor of CP, along with its higher prevalence in the L-NAFLD population, it can be inferred that genetic diversity plays a role in the correlation between L-NAFLD and CP.

2) Sarcopenia is an age-related decline in muscle mass and function [32], while sarcopenic obesity refers to the gradual accumulation of fat with muscle loss [33]. Individuals with FLD have a higher risk of ASCVD when transitioning from non-sarcopenia to sarcopenia, with the risk increasing from 1.47 to 4.08, alongside increasing the substantial risk of advanced liver fibrosis (adjusted OR = 2.48 by FIB-4) [34]. Meanwhile, the link between L-NAFLD and sarcopenic obesity is even more intimate, regardless of metabolic factors [35], cause compared to non-lean NAFLD individuals, L-NAFLD population tend to have less severe obesity and potentially reduced muscle mass [28], and then a potentially higher prevalence of sarcopenic obesity, which can be worsen by IR, an independent risk factor for ASCVD [25].

Another highlight of our research, is the exploration of cutoff values for risk factors, which is rarely mentioned in other studies. Despite SBP, FBG and LDL-c were identified as independent risk factors for CP and consistent with previous research [36], the surprising finding was that their cutoff values were lower than normal upper limits. We fully acknowledge the comprehensive investigations conducted to elucidate the underlying mechanisms associated with these risk factors [10,37], while our finding prompts us to re-consider the L-NAFLD. This population may represent as high-risk subgroup base on our and previous finding [24,38,39], implementing stringent management objectives could potentially reduce their susceptibility to developing advanced hepatic disorders and associated complications, similar to the current guidelines for type 2 diabetes and dyslipidemia in China [40,41]. Nonetheless, further research is imperative to validate this hypothesis, and our findings perhaps serve as a valuable reference.

Our research has limitations. First, the lack of information regarding smoking, medical history, medication usage, and other pertinent factors poses a challenge to our analysis of more crucial variables; the inadequacy of measurements such as waist circumference, body composition analysis, and fasting insulin level hinders further investigation into the role of abdominal obesity in NAFLD and metabolic dysfunction-associated steatotic liver diseases (MASLD), a newly coined term for NAFLD [42]; the unavailability of FibroTouch or FibroScan prevents early-stage fatty liver patient screening [43]. Second, this study is limited to a single center, so more multi-center studies across diverse regions are needed to consider factors like geographical location, race, and lifestyle that may influence the results. Thirdly, this retrospective study lacks longitudinal follow-up, thereby impeding the establishment of a causal relationship between NAFLD and CP, and limiting our comprehension of prognosis across diverse BMI populations with NAFLD.

## Conclusion

The association between NAFLD and CP appears to be more prominent in lean individuals, lean individuals present with CP may experience more severe liver injury, and implementing stricter health management criteria may benefit their health conditions.

## Supporting information

S1 Table Baseline characteristics of 4 groups and comparison in lean and non-lean population. (DOCX)

S1 Data (XLSX)

S2 Data (XLSX)

## Abbreviations

ALT: alanine aminotransferase
ASCVD: atherosclerotic cardiovascular disease
AST: aspartate aminotransferase
AUC: area under the curve
BMI: body mass index
BUN: blood urea nitrogen
CI: confidence interval
CP: carotid plaque
CRE: creatinine
CT: computerized tomography
DBP: diastolic blood pressure
FBG: fasting blood glucose
FIB-4: Fibrosis-4 index
GGT: gamma-glutamyl transferase
HB: hemoglobin
HDL-c: high density lipoprotein cholesterol
IMT: intima-media thickness
IQR: interquartile range
L-NNAFLD: lean but absent with NAFLD
MRI: magnetic resonance imaging
NAFLD: non-alcoholic fatty liver disease
OR: odds ratio
PLT: platelet count
ROC: receiver operating characteristic
SBP: systolic blood pressure
TC: total cholesterol
TG: triglyceride
UA: uric acid
