# Supplementary material for: Higher risk of carotid plaque among lean individuals with non-alcoholic fatty liver disease: A retrospective study
Source: PLoS One. 2025 Feb 3;20(2):e0316997. doi: 10.1371/journal.pone.0316997 (PMC11790118; doi:10.1371/journal.pone.0316997)
Supplement: S1 Table — (DOCX) [file pone.0316997.s001.docx]

# Supplement table 1. Baseline characteristics of 4 groups and comparison in lean and non-lean population

**Supplement table 1. Baseline characteristics of 4 groups and comparison in lean and non-lean population**

| Characteristics | Total (3587) | Lean | | | Non-lean | | |
| --- | --- | --- | --- | --- | --- | --- | --- |
|  |  | CP [735(20.5)] | NCP [848(23.6)] | *Z/χ^2^* (*P*) | CP [965(26.9)] | NCP [1039(29.0)] | *Z/χ^2^* (*P*) |
| Demographics |  |  |  |  |  |  |  |
| Age (year) | 56 (15) | 61 (15) | 52 (13) | -17.945 (**<0.001^a^**) | 60 (17) | 52 (12) | -18.250 (**<0.001**) |
| 18~39 [*n* (*%*)] | 247 (6.9) | 10 (1.4) | 112 (13.2) | 249.778 (**<0.001**) | 13 (1.3) | 112 (10.8) | 285.295 (**<0.001**) |
| 40~64 [*n* (*%*)] | 2468 (68.8) | 415 (56.4) | 646 (76.2) | NA | 577 (59.8) | 830 (79.9) | NA |
| 65 [*n* (*%*)] | 872 (24.3) | 310 (42.2) | 90 (10.6) | NA | 375 (38.9) | 97 (9.3) | NA |
| Gender [*n* (*%*)] |  |  |  | 30.912 (**<0.001**) |  |  | 2.650 (0.104) |
| Male | 2023 (56.4) | 393 (53.5) | 335 (39.5) | NA | 641 (66.4) | 654 (62.9) | NA |
| Female | 1564 (43.6) | 342 (46.5) | 513 (60.5) | NA | 324 (33.6) | 385 (37.1) | NA |
| Anthropometric measure |  |  |  |  |  |  |  |
| Height (cm) | 162.0 (12.4) | 160.5 (13.0) | 160.0 (11.0) | -0.656 (0.512) | 162.5 (11.8) | 163.5 (12.5) | -2.251 (**0.024**) |
| Weight (Kg) | 64.0 (15.6) | 56.0 (11.3) | 56.00 (9.8) | -0.926 (0.355) | 70.0 (12.4) | 71.0 (13.3) | -2.365 (**0.018**) |
| SBP (mmHg) | 130 (23) | 132 (24) | 121 (24) | -11.611 (**<0.001**) | 136 (24) | 129 (20) | -9.729 (**<0.001**) |
| DBP (mmHg) | 78 (16) | 76 (15) | 74 (15) | -4.696 (**<0.001**) | 80 (15) | 80 (16) | -1.055 (0.291) |
| BMI (Kg/m^2^) | 24.4 (4.1) | 22.23 (2.30) | 22.26 (2.20) | -0.677 (0.498) | 26.26 (2.71) | 26.36 (2.82) | -0.998 (0.318) |
| Serological tests |  |  |  |  |  |  |  |
| PLT (×10^9^/L) | 199.2 (66.9) | 195.3 (67.7) | 202.0 (63.7) | -1.78 (0.075) | 195.6 (66.6) | 203.5 (70.4) | -3.815 (**<0.001**) |
| HB (g/L) | 141.2 (19.6) | 138.1±13.6 | 136.6 (18.1) | -1.196 (0.232) | 143.4 (18.7) | 146.0 (19.7) | -3.323 (**<0.001**) |
| ALT (U/L) | 20.0 (12.6) | 16.9 (9.5) | 17.1 (10.3) | -0.270 (0.787) | 21.4 (12.8) | 23.7 (16.6) | -5.507 (**<0.001**) |
| AST (U/L) | 22.7 (7.2) | 22.7 (6.7) | 21.9 (6.8) | -2.715 (**0.007**) | 22.7 (7.0) | 23.6 (8.2) | -2.578 (**0.010**) |
| GGT (U/L) | 24.4 (22.3) | 20.9 (16.1) | 19.2 (14.2) | -3.600 (**<0.001**) | 28.6 (23.6) | 29.9 (26.8) | -1.346 (0.178) |
| TG (mmol/L) | 1.41 (1.03) | 1.19 (0.76) | 1.15 (0.74) | -1.718 (0.086) | 1.6 (1.1) | 1.7 (1.2) | -1.551 (0.121) |
| TC (mmol/L) | 4.9 (1.3) | 5.0±1.0 | 4.8 (1.1) | -2.904 (**0.004**) | 5.0±1.0 | 4.9 (1.3) | -0.667 (0.505) |
| HDL-c (mmol/L) | 1.6 (0.5) | 1.7 (0.5) | 1.7 (0.6) | -0.708 (0.479) | 1.5 (0.5) | 1.5 (0.5) | -0.255 (0.798) |
| LDL-c (mmol/L) | 2.59 (0.88) | 2.61±0.70 | 2.45 (0.79) | -3.683 (**<0.001**) | 2.69±0.70 | 2.63 (0.79) | -0.700 (0.484) |
| FBG (mmol/L) | 5.31 (0.89) | 5.28 (0.92) | 5.08 (0.68) | -8.063 (**<0.001**) | 5.54 (1.24) | 5.36 (0.83) | -6.791 (**<0.001**) |
| CRE (μmol/L) | 67.9 (21.3) | 66.3 (20.4) | 63.0 (19.7) | -3.877 (**<0.001**) | 70.9 (20.1) | 70.4 (21.6) | -1.562 (0.118) |
| BUN (mmol/L) | 5.3 (1.8) | 5.5 (1.9) | 5.2 (1.7) | -4.873 (**<0.001**) | 5.4 (1.8) | 5.3 (1.7) | -3.947 (**<0.001**) |
| UA (μmol/L) | 336.2 (121.7) | 312.9 (104.9) | 304.1 (112.0) | -1.536 (0.125) | 356.8 (121.4) | 362.7 (124.3) | -0.882 (0.378) |
| FIB-4 | 1.41 (0.90) | 1.72 (0.99) | 1.34 (0.81) | -10.804 (**<0.001**) | 1.55 (0.98) | 1.24 (0.71) | -11.604 (**<0.001**) |
| ＜1.30 [ *n* (*%*)] | 1485 (41.4) | 183 (24.9) | 399 (47.1) | 103.985 (**<0.001**) | 335 (34.7) | 568 (54.7) | 118.046 (**<0.001**) |
| 1.30~2.67 [ *n* (*%*)] | 1796 (50.1) | 439 (59.7) | 404 (47.6) | NA | 511 (53.0) | 442 (42.5) | NA |
| ＞2.67~＜3.48 [ *n* (*%*)] | 209 (5.8) | 72 (9.8) | 32 (3.8) | NA | 82 (8.5) | 23 (2.2) | NA |
| ≥3.48 [ *n* (*%*)] | 97 (2.7) | 41 (5.6) | 13 (1.5) | NA | 37 (3.8) | 6 (0.6) | NA |
| NAFLD [ *n* (*%*)] |  |  |  | 13.673 (**<0.001**) |  |  | 0.096 (0.757) |
| Presence | 1877 (52.3) | 223 (30.3) | 188 (22.2) | NA | 709 (73.5) | 757 (72.9) | NA |
| Absence | 1710 (47.7) | 512 (69.7) | 660 (77.8) | NA | 256 (26.5) | 282 (27.1) | NA |

^a^The bold numbers signify statistical significance.

BMI: body mass index; Lean: BMI ＜24 Kg/m^2^; non-Lean: BMI≥24 Kg/m^2^; CP: carotid plaque; NCP: absent with CP; SBP: systolic blood pressure; DBP: diastolic blood pressure; PLT: platelet count; HB: hemoglobin; ALT: alanine aminotransferase; AST: aspartate aminotransferase; GGT: gamma-glutamyl transferase; TG: triglyceride; TC: total cholesterol; HDL-c: high density lipoprotein cholesterol; LDL-c: low density lipoprotein cholesterol; FBG: fasting blood glucose; CRE: creatinine; BUN: blood urea nitrogen; UA: uric acid; FIB-4: Fibrosis-4 index; NAFLD: non-alcoholic fatty liver disease.
